# Surgical Complexity and Outcome During the Implementation Phase of a Robotic Colorectal Surgery Program—A Retrospective Cohort Study

**DOI:** 10.3389/fonc.2020.603216

**Published:** 2021-02-16

**Authors:** Catharina Müller, Johannes Laengle, Stefan Riss, Michael Bergmann, Thomas Bachleitner-Hofmann

**Affiliations:** Department of Surgery, Division of General Surgery, Comprehensive Cancer Center Vienna, Medical University of Vienna, Vienna, Austria

**Keywords:** robotic surgery, colorectal cancer, colorectal surgery, case complexity, learning curve, DaVinci Si

## Abstract

**Background:**

Robotic surgery holds particular promise for complex oncologic colorectal resections, as it can overcome many limitations of the laparoscopic approach. However, similar to the situation in laparoscopic surgery, appropriate case selection (simple vs. complex) with respect to the actual robotic expertise of the team may be a critical determinant of outcome. The present study aimed to analyze the clinical outcome after robotic colorectal surgery over time based on the complexity of the surgical procedure.

**Methods:**

All robotic colorectal resections (n = 85) performed at the Department of Surgery, Medical University of Vienna, between the beginning of the program in April 2015 until December 2019 were retrospectively analyzed. To compare surgical outcome over time, the cohort was divided into 2 time periods based on case sequence (period 1: patients 1–43, period 2: patients 44–85). Cases were assigned a complexity level (I-IV) according to the type of resection, severity of disease, sex and body mass index (BMI). Postoperative complications were classified using the Clavien-Dindo classification.

**Results:**

In total, 47 rectal resections (55.3%), 22 partial colectomies (25.8%), 14 abdomino-perineal resections (16.5%) and 2 proctocolectomies (2.4%) were performed. Of these, 69.4% (n = 59) were oncologic cases. The overall rate of major complications (Clavien Dindo III-V) was 16.5%. Complex cases (complexity levels III and IV) were more often followed by major complications than cases with a low to medium complexity level (I and II; 25.0 vs. 5.4%, p = 0.016). Furthermore, the rate of major complications decreased over time from 25.6% (period 1) to 7.1% (period 2, p = 0.038). Of note, the drop in major complications was associated with a learning effect, which was particularly pronounced in complex cases as well as a reduction of case complexity from 67.5% to 45.2% in the second period (p = 0.039).

**Conclusions:**

The risk of major complications after robotic colorectal surgery increases significantly with escalating case complexity (levels III and IV), particularly during the initial phase of a new colorectal robotic surgery program. Before robotic proficiency has been achieved, it is therefore advisable to limit robotic colorectal resection to cases with complexity levels I and II in order to keep major complication rates at a minimum.

## Introduction

The introduction of minimally invasive surgery has led to faster recovery and better short- and long-term outcomes. Thus, laparoscopy has become a well-established approach across all surgical fields. For colorectal surgery, laparoscopic bowel resection has become standard of care and its efficacy and safety has also been proven for oncologic resections (1–4). However, laparoscopic rectal resection is limited through the confined working space. Therefore, laparoscopic rectal surgery has a slow learning curve and is still associated with high rates of conversion to open surgery (5). Technical advantages of the robotic surgery system such as stable 3D vision with a camera that can constantly be adjusted by the surgeon himself, an extended range of motion of the instruments and better ergonomics may overcome drawbacks of laparoscopy in complex cases and narrow operation spaces such as the pelvis. Accordingly, to date robotic surgical systems are more often used for rectal than colonic surgery (6). Additionally, it has recently been shown that the robotic approach could be especially favorable in high-risk subgroups such as male or obese patients or patients with locally advanced tumors that have undergone preoperative radiation therapy (7). The robotic approach may also lead to a better functional outcome, lower conversion rates and a higher histopathologic quality of the resected specimen, with an enhanced completeness of total mesorectal excision (TME) and an improved circumferential resection margin (CRM) (7–9).

However, similar to the situation in laparoscopy, experience of the surgeon is essential for the success of robotic surgery. To overcome increased complication rates in the learning phase, a complexity score was proposed for laparoscopic colorectal resection to minimize surgical risk in the beginning (10). The situation in robotic surgery could be similar: Even though complex cases may especially benefit from robotic surgery, appropriate case selection with respect to the actual experience in robotic surgery may still be a critical determinant of outcome, especially in the implementation phase of this new operation technique (11).

The aim of our study was to describe our learning curve and implementation phase of robotic colorectal surgery at the Medical University of Vienna and to analyze the outcome in robotic surgery over time based on the complexity of surgery.

## Material and Methods

All patients (n = 85), who underwent robotic large bowel resection at a single tertiary referral center, the Medical University of Vienna, from the start of the robotic surgery program in April 2015 until December 2019, were included in the present retrospective cohort study. Patients who qualified for a minimally invasive approach were preferentially considered for robotic surgery. However, the actual decision whether to perform a procedure using the robotic or laparoscopic approach largely depended on the availability of the robot since the latter was shared between the Departments of Urology, Surgery and Gynaecology at our hospital. Priority was given to rectal resections over left and right-sided colonic resections due to a perceived better cost-benefit ratio of pelvic robotic surgery. Furthermore, oncologic resections were given priority over benign/inflammatory conditions. A team of two experienced colorectal surgeons (T.B.-H. and M.B.), both with a previous laparoscopic experience of >100 colorectal resections performed all surgeries with the robotic surgery system DaVinci^®^ Si (Intuitive Surgical, Inc., Sunnyvale CA, USA). Robotic training was performed as follows: After initial hands-on training in a pig model, one proctored cholecystectomy was performed before the team started with robotic colorectal resections. Both surgeons enrolled in the European Academy of Robotic Colorectal Surgery (EARCS) training program. In the majority of cases, both robotic surgeons were operating simultaneously, each of them from one of two DaVinci^®^ consoles available at our institution. A standardized set-up was implemented with the same port placement and sequence of surgical steps for each individual procedure. For rectal and left colonic resections, a medial-to-lateral approach was applied to dissect the vessels and mobilize the left colonic flexure. Rectal resections were performed according to the principles of total mesorectal excision using a pelvic set-up of the robotic arms as described previously (12). Specimens were always retrieved *via* a supra-pubic incision. The rectal anastomosis was performed under direct laparoscopic vision and using a circular stapler. In right-sided resections, the specimen was retrieved using a periumbilical midline incision. The anastomotic technique consisted of a side-to-side stapled anastomosis performed extracorporeally.

Data were retrospectively retrieved from our institutional database and individual patient charts. The study was approved by the local ethics committee (EK 1639/2020). The collected baseline characteristics consisted of age, sex, body mass index (BMI), American Society of Anaesthesiologists (ASA) classification, smoking habits and co-morbidities. Surgical data consisted of indication for surgery, type of surgical procedure and stoma formation. Conversion was defined as an extension of the initially planned incision.

The study cohort was divided into two time periods based on the chronological case sequence to compare surgical outcome over time (period 1: cases 1–43; period 2: cases 44–85). This categorization with patient 44 as a cut-off point was based on a marked drop of the complication rate after case 44 of our series. The EARCS training phase was part of period 1 and consisted of the first 30 cases. The primary outcome parameter was surgical complications within 30 days from the primary surgery. Surgical complications were classified according to the Clavien Dindo Classification and subsequently divided into minor (Clavien Dindo I and II) and major complications (Clavien Dindo III-V) (13). Lymph node (LN) retrieval, operation time and length of hospital stay were assessed as further indicators of outcome. Operative time was calculated from the moment of the first skin incision until the end of skin closure and included all robot docking and undocking times as well as any additional surgical procedures such as laparoscopic stoma formation at the end of surgery.

Each case was assigned a complexity level using the complexity score by Miskovic et al. for laparoscopic surgery (10). The complexity score includes four levels (I-IV) according to type of resection, underlying disease (cancer vs. inflammatory), BMI and sex. The score is presented in detail in **Supplemental Figure 1**. Importantly, the score has previously also been used in the context of robotic surgery (11). For statistical analysis, cases with complexity levels I/II were defined as “low complexity group” while cases with complexity levels III/IV were defined as “high complexity group”.

### Statistical Analysis

IBM Statistical Package for the Social Sciences (SPSS) Version 24 for Mac (SPSS Inc., Chicago, IL, USA) was used for statistical analysis. Descriptive data for continuous variables were reported as mean and standard deviation or median and interquartile range (IQR) as applicable. Categorical parameters were given in numbers and percentages. Student’s t-test was performed to calculate group differences for continuous parameters if normally distributed and the absence of outliers. Otherwise, the Wilcoxon-Mann-Whitney U-Tests was applied. We used Pearson χ^2^-test for categorical variables. A significance level of a two sided p-value ≤ 0.05 was considered statistically significant.

## Results

### Baseline Characteristics and Short-Term Outcome

**Tables 1** and **2** show the detailed demographic and surgical data for our patient cohort. In total, 47 rectal resections (55.3%), 22 partial colectomies (25.8%), 14 abdomino-perineal resections (APR, 16.5%) and 2 proctocolectomies (2.4%) were performed. Overall 69.4% of patients (n = 59) had an uneventful postoperative course, while 14.1% (n = 12) developed minor and 16.5% (n = 14) developed major complications. Major complications consisted of anastomotic leak (n = 3, 3.5%), ischemia of the neorectum (n = 3, 3.5%), compartment syndrome (n = 3, 3.5%), mechanical ileus due to internal (n = 1, 1.2%) or parastomal hernia (n = 1, 1.2%), pelvic abscess (n = 1, 1.2%), ureteral leak (n = 1, 1.2%) and acute respiratory distress syndrome (ARDS) (n = 1, 1.2%). The conversion rate to open surgery was 5.9% (n = 5). The reasons for conversion were anatomical difficulties due to adhesions (n = 3), small bowel injury (n = 1) and persistent bradycardia following establishment of the pneumoperitoneum (n = 1). Forty patients (47.1%) received a protective stoma. The median length of hospital stay was 8.5 days (IQR, 7–13d) and the 30-day readmission rate was 4.7% (n = 4).

**Table 1:** Baseline characteristics.

	Overalln = 85	Period 1n = 43	Period 2n = 42	
**Diagnosis**				p = 0.943
Cancer	59 (69.4)	30 (69.8)	29 (69.0)	
Inflammatory	26 (30.6)	13 (30.2)	13 (31.0)	
**Age**				p = 0.231
(years)	58.0 ( ± 13.5)	59.7 ( ± 12.9)	56.2 ( ± 14.0)	
**Sex**				p = 0.011*
Male	52 (61.2)	32 (74.4)	20 (47.6)	
Female	33 (38.8)	11 (25.6)	22 (52.4)	
**BMI**				p = 0.073
(kg/m^2^)	24.8 (22.2–29.1)	26.7 (23.0–29.4)	23.8 (21.6–27.8)	
**ASA classification**				p = 0.540
Grade I	9 (10.6)	3 (7.0)	6 (14.3)	
Grade II	52 (61.2)	27 (62.8)	25 (59.5)	
Grade III	24 (28.2)	13 (30.2)	11 (26.2)	
**Diabetes**				p = 0.563
No	73 (85.9)	36 (83.7)	37 (88.1)	
Yes	12 (14.1)	7 (16.3)	5 (11.9)	
**Smoking**				p = 0.411
No	61 (71.8)	29 (67.4)	32 (76.2)	
Yes	17 (20.0)	10 (23.3)	7 (16.7)	
Unknown	7 (8.2)	4 (9.3)	3 (7.1)	

*significant at <0.05 level.

**Table 2:** Surgical data.

	Overalln = 85	Period 1n = 43	Period 2n = 42	
**Procedure**				p = 0.523
Partial colectomy	22 (25.9)	11 (25.6)	11 (26.2)	
Rectal resection	47 (55.3)	24 (55.8)	23 (54.8)	
APR^◆^	14 (16.5)	6 (14.0)	8 (19.0)	
Proctocolectomy	2 (2.4)	2 (4.7)	–	
**Stoma formation**				p = 0.229
No	45 (52.9)	20 (46.5)	25 (59.5)	
Yes	40 (47.1)	23 (53.5)	17 (40.5)	
**Complexity level**				p = 0.172
Level I	11 (12.9)	3 (7.0)	8 (19.0)	
Level II	26 (30.6)	11 (25.6)	15 (35.7)	
Level III	32 (37.6)	19 (44.2)	13 (31.0)	
Level IV	16 (18.8)	10 (23.3)	6 (14.3)	
**Complexity level**				p = 0.039*
Simple	37 (43.5)	14 (32.6)	23 (54.8)	
Complex	48 (56.5)	29 (67.4)	19 (45.2)	
**Operation time** (min)	350 ( ± 93)	376 ( ± 93)	323 ( ± 87)	p = 0.009*
**Conversion**				p =1.000
Yes	5 (5.9)	3 (7.0)	2 (4.8)	
No	80 (94.1)	40 (93.0)	40 (95.2)	
**Major complication**				p = 0.038*
Yes	14 (16.5)	11 (25.6)	3 (7.1)	
No	71 (83.5)	32 (74.4)	39 (92.9)	
**Length of stay** (days)	8.5 (7–13)	10 (8–16)	7 (6–10)	p = 0.001*
**Harvested lymph nodes**	13 (10–19)	13 (10–19)	13 (11–23)	p = 0.342

^◆^Abdominoperineal resection, *significant at <0.05 level.

### Complexity Level and Risk Factors for Complications

**Figure 1:**
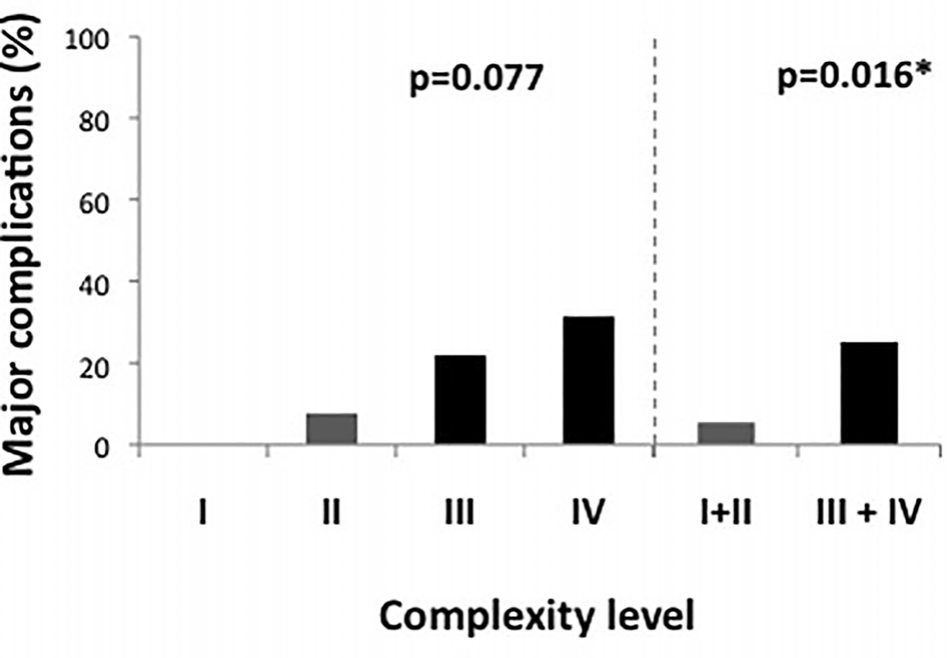
Major complications according to complexity score. **Figure 1** shows complexity scores in detail on the left side and divided in simple and complex cases (levels I and II vs. levels III and IV) on the right side of the graph. Complex cases had a significantly higher rate of major complications. *significant at 0.05 level.

There were 52 male (61.2%) and 33 female (38.8%) patients with a median BMI of 24.8 kg/m^2^ (IQR, 22.2–29.1 kg/m^2^). Bowel resection was performed for malignancy in 59 patients (69.4%), 23 of whom had preoperative radiation therapy (39%). Based on these data, 12.9% of cases (n = 11) were classified as complexity level I, 30.6% as level II (n = 26), 37.6% as level III (n = 32) and 18.8% as level IV (n = 16), yielding a total of 37 simple (levels I + II combined, 43.5%) and 48 complex (levels III + IV combined, 56.5%) cases. As shown in **Figure 1**, there were no major complications in cases with level I complexity, while the rates of major complications increased to 7.7%, 21.9% and 31.1% in patients with complexity levels II, III, and IV, respectively (p = 0.077). Taken together, cases with high complexity (levels III + IV) showed a significantly higher rate of major complications than cases with low complexity (levels I + II) (25.0 vs. 5.4%, p = 0.016). In patients with high case complexity, operation time was significantly longer than in patients with low case complexity (417 ± 67 vs. 337 ± 92 min, p =0.003). Additionally, the conversion rate to open surgery was 10.4% in patients with high complexity, while there were no conversions in the group of patients with low complexity. This difference was statistically significant (p = 0.043, data not shown).

The major complication rate for male patients was 21.2% (n = 11/52) while it was only 9.1% of female patients (n = 3/33). However, the difference did not reach statistical significance (p = 0.144, data not shown).

### Complexity Level and Outcome Over Time

**Figure 2:**
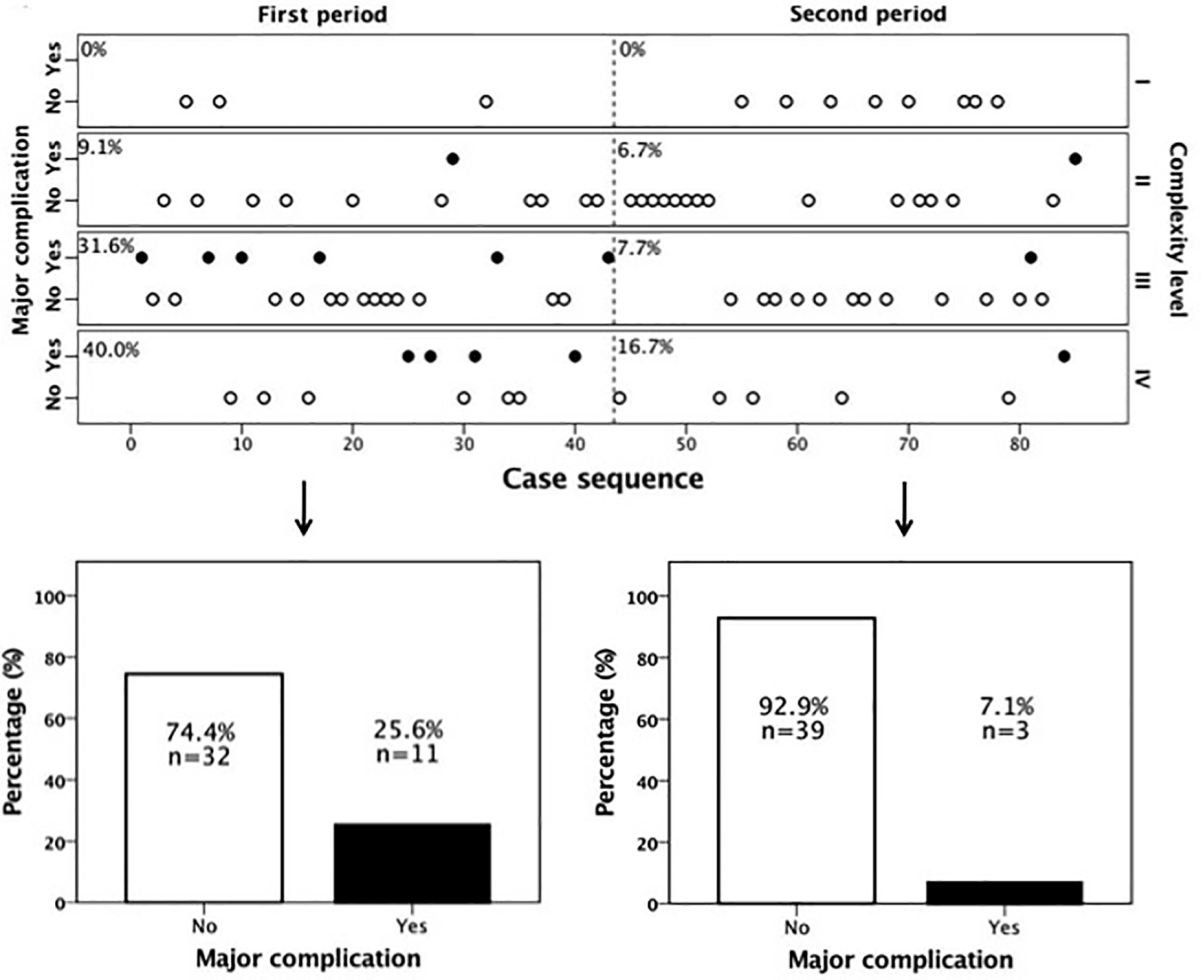
Major complications over time according to case complexity. There were significantly more major complications in the first time period. From case 44 onwards the occurrence of major complications declined markedly.

During the first time period (cases 1–43), 67.4% (29/43) of the cases were of high complexity, while this percentage dropped significantly to 45.2% (19/42) in the second period (cases 44–85) (p = 0.039, **Table 2**). Reflecting the higher case complexity in the initial phase of the program, operation time dropped significantly from 376 ± 93min in the first period to 323 ± 87min in the second period (p = 0.009). However, the oncologic quality of resection, reflected by the amount of harvested lymph nodes, remained identical (median, 13 LN; IQR, 10–19 (first time period) vs. median, 13 LN; IQR, 10–23 (second time period), p = 0.342). **Figure 2** shows all individual cases sorted in chronological order and according to their complexity level to allow for a more detailed analysis of the occurrence of major complications over time as well as their association with complexity level. It becomes evident that there were not only significantly more complex cases but also significantly more major complications in the first as compared to the second period (25.6 vs. 7.1%, p = 0.038). This was also reflected by a significantly longer hospital stay in the first as compared to the second time period (10 days (IQR, 8–16d) vs. 7 days (IQR, 6–10d), p = 0.001).

**Figure 3:**
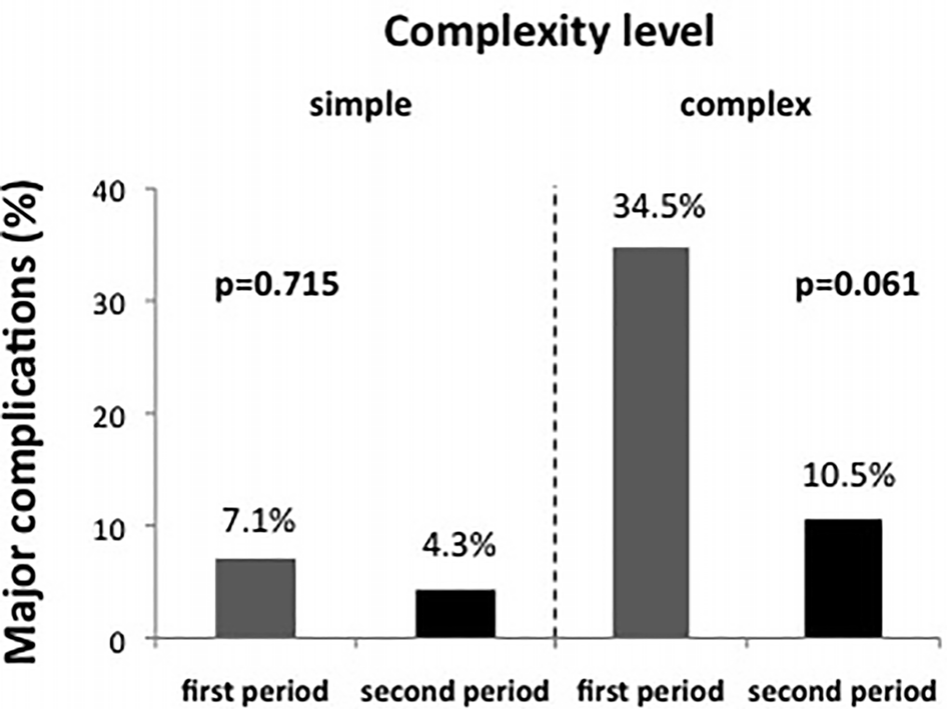
The evolution of major complication rate over time for simple and complex cases separately. The reduction of major complication over time was particularly pronounced in complex cases, even though in neither group a statistically significant difference was noted.

To assess whether the reduction in complication rate in the second period was primarily due to the lower case complexity or whether an additional learning effect could be observed, we subsequently performed a combined analysis taking both case complexity and time period into account. As shown in **Figure 3**, patients with high complexity had a major complication rate of 34.5% (10/29 patients) in the first time period, which dropped to 10.5% (2/19 patients) in the second time period. At the same time, the major complication rate in the group of patients with low complexity dropped from 7.1% (1/14) to 4.3% (1/23). Even though the reduction of major complications in neither patient group was statistically significant, the reduction of major complications in patients with high complexity was more pronounced (-69.6% relative risk reduction, p = 0.061) than in patients with low complexity (-39.4% relative risk reduction, p = 0.715).

## Discussion

Our study shows that the Miskovic complexity score correlates with the rate of major complications in robotic colorectal surgery. Major complication rates increased from 0% in cases with complexity level I to 7.7, 21.9, and 31.1% in levels II, III and IV, respectively. Importantly, a learning effect during the first study period, which was particularly pronounced in complex cases, in combination with a reduced case complexity in the second study period, led to a significant reduction of major complications over time (25.6 vs. 7.1%, p = 0.038). Our results suggest that the Miskovic complexity score could be a valuable tool to guide colorectal robotic surgeons in choosing clinical cases that are appropriate to their actual robotic experience, thus keeping the rate of major complications low. This is especially relevant during the early implementation phase of a new colorectal robotic program as well as in oncologic patients, who often require complex surgery and are in particular need of a fast and uncomplicated postoperative recovery.

Recent data suggest an advantage of robotic over laparoscopic surgery in patients undergoing complex colorectal surgery (e.g. male patients with low rectal cancer) (14). Several authors have demonstrated that robotic surgery has a short learning curve, which seems to be independent from previous laparoscopic experience (15–20). Moreover, with structured training programs such as the curriculum of the EARCS available (12, 21, 22), case series with virtually no complications during the implementation phase of robotic surgery have been published (22, 23). Thus, the risks associated with implementing a new colorectal robotic program are perceived as minimal and the resulting enthusiasm for robotic surgery has led multiple teams in different hospital settings to rapidly introduce robotic surgery into clinical routine.

However, robotic surgery is expensive, which limits the number of hospitals offering it. Moreover, robotic systems are often used by multiple disciplines, hindering access to the robotic platform. As a result, many surgeons narrow the indication spectrum for robotic surgery, giving priority to patients requiring complex surgery (who are supposed to derive an increased benefit from the robotic approach such as male patients with low rectal cancer), thereby also trying to increase cost-effectiveness. When we started our colorectal robotic program in April 2015, the robot was at our disposal for one day per week. In view of the above considerations, our institutional decision was to give priority to patients requiring oncologic surgery to the rectum and left colon over patients with right-sided colon cancer or benign conditions. This resulted in 67.4% (29/43) of the initial surgeries in our cohort being of high complexity. Somewhat unexpectedly and in contrast to many other reports, we observed a high rate of major complications during the early phase of our program. Of note, major complications occurred almost exclusively in patients with high case complexity. Therefore, we conclude that the case-complexity during the initial phase of our program exceeded our level of robotic expertise at that time. However, apart from this higher postoperative complication rate at the beginning of our program, the quality of the surgical specimens as assessed by the number of retrieved lymph nodes was satisfactory from the beginning and did not differ significantly over time.

Importantly, there was a sharp decline of major complications from patient 44 onward, which was particularly pronounced in patients with high complexity, indicating a learning effect. However, we also observed a reduced case complexity in the second half of our study, which most likely reflected a more defensive patient selection in view of the high complication rate in the initial phase of our program. In our opinion, both factors contributed to the significant reduction of the major complication rate observed in the second phase of our study (25.6 vs. 7.1%, p = 0.038).

Several factors may account for the worse outcomes of our study as compared to previous reports: First, even though conclusions on case complexity from other studies can only be rough estimates, we feel that the case complexity in our study (67.4% in the initial phase, 56.5% overall) was significantly higher than in other series. For example, in the cohort reported by Aradaib et al., who reported an extremely low complication rate in their paper on the safe adoption of colorectal robotic surgery in Ireland, only 40% (22/55) of patients had rectal cancer or complicated diverticular disease suggestive of higher surgical complexity (23). Second, we used a DaVinci^®^ Si-system, which is known to be less suited for multi-quadrant surgery (an integral component of left-sided colonic or rectal resections). It is thus more prone to intraoperative difficulties and longer operation times than the more recently developed DaVinci^®^ Xi-system, which can easily navigate between different quadrants of the abdomen without the need of re-docking or inadvertent arm collisions (22). Third, both lead surgeons started their robotic training at the same time, resulting in superimposing learning curves in the very beginning, which may have hindered a faster progression of the learning process in our team. A consecutive learning approach may have been more advisable as it has been shown that the number of procedures needed to reach proficiency in colorectal robotic surgery drops from 75 operations for the first robotic surgeon of a new program to about 25–30 procedures for all subsequent surgeons (24).

Our report should be taken as an honest word of caution and a pledge for a slow and structured implementation of colorectal robotic surgery into clinical practice. Important achievements of the robotic community such as the structured EARCS training program are already available and have largely standardized robotic surgery education in a formidable way. However, we feel that we are still missing a standardized successive implementation pathway, which could help emerging robotic teams to implement their new programs more efficiently and safely. In this regard, the Miskovic complexity score could be an important selection tool. In view of the fact that we saw hardly any complications in patients with low complexity (levels I and II according to the Miskovic score) even in the very early phase of our program, it would seem reasonable to start a program exclusively with cases of this complexity level. Thereafter, the case complexity could be gradually increased to level III and IV surgeries, depending on the actual complication rates and expertise of the team. Miskovic et al. proposed a stepwise approach based on case complexity for laparoscopic surgery and suggested to perform about 50 cases of each complexity level before moving on to the next level (10). This stepwise approach seems equally feasible in robotic colorectal surgery. Exact numbers needed for each complexity level remain to be defined in future studies, but in view of the marked drop of major complications in our study from patient 44 onward, a learning curve of approximately 40 cases until robotic proficiency seems realistic.

There are several limitations of our study: It is a retrospective, single center analysis of the implementation of a robotic colorectal surgery program where the outcome of the whole team and not the results of individual surgeons have been analyzed. Only the DaVinci® Si system was used for robotic surgery, which is less suited for multi-quadrant abdominal surgery than the next generation Xi-system. Furthermore, due to sample size and study design the applicability of the complexity score in the implementation phase of colorectal robotic surgery programs has yet to be validated in further prospective trials.

In conclusion, the Miskovic complexity score represents a promising tool for patient selection in the learning phase of robotic colorectal surgery. Simple cases can be performed with a minimal risk of major of complications even in the very early phase of a new program, while complex cases should not be taken on before proficiency in simple cases has been achieved. Thus, not only case-load but also case complexity should be considered as key factors for the safe implementation of robotic surgery into clinical practice. This is of particular importance in patients with oncologic resections who often require complex surgery and derive particular benefit from minimal postoperative morbidity. A dedicated surgical team, a standardization of the surgical set-up and procedural steps as well as a standardization of perioperative patient care (such as enhanced recovery after surgery, ERAS) altogether form the basis of a successful colorectal robotic surgery program. However, we feel that particularly in the very early phases of a new program, appropriate patient selection seems to be one of the most crucial factors to influence perioperative outcome of colorectal robotic surgery.

## Data Availability Statement

The raw data supporting the conclusions of this article will be made available by the authors, without undue reservation.

## Ethics Statement

The studies involving human participants were reviewed and approved by Ethics Committee of the Medical University of Vienna, Austria, EK 1639/2020. Written informed consent for participation was not required for this study in accordance with the national legislation and the institutional requirements.

## Author Contributions

CM: Contribution to the study concept design, data acquisition, analysis and interpretation of data, writing the first draft, and final approval. JL: Data acquisition, revision of scientific content, and final approval. SR: Data acquisition, revision of scientific content and final approval. MB: Contribution to study concept, data acquisition, revision of scientific content, and final approval. TB-H: Scientific supervisor, contribution to the study concept design, acquisition and interpretation of data, correction and critical revision of the draft, and final approval. All authors contributed to the article and approved the submitted version.

## Conflict of Interest

The authors declare that the research was conducted in the absence of any commercial or financial relationships that could be construed as a potential conflict of interest.

## Abbreviations

APR, Abdomino-perineal resection; ASA, American Society of Anaesthesiologists; CRM, circumferential resection margin; BMI, body mass index; EARCS, European Academy of Robotic Colorectal Surgery; IQR, interquartile range; LN, lymph node; TME, total mesorectal excision.

## References

[1] Guillou PJ Quirke P Thorpe H Walker J Jayne DG Smith AM. Short-term endpoints of conventional versus laparoscopic-assisted surgery in patients with colorectal cancer (MRC CLASICC trial): multicentre, randomised controlled trial. Lancet (2005) 365(9472):1718–26. 10.1016/S0140-6736(05)66545-2 15894098

[2] Jayne DG Thorpe HC Copeland J Quirke P Brown JM Guillou PJ. Five-year follow-up of the Medical Research Council CLASICC trial of laparoscopically assisted versus open surgery for colorectal cancer. Br J Surg (2010) 97(11):1638–45. 10.1002/bjs.7160 20629110

[3] van der Pas MH Haglind E Cuesta MA Fürst A Lacy AM Hop WC. Laparoscopic versus open surgery for rectal cancer (COLOR II): short-term outcomes of a randomised, phase 3 trial. Lancet Oncol (2013) 14(3):210–8. 10.1016/S1470-2045(13)70016-0 23395398

[4] Araujo SE Seid VE Klajner S. Robotic surgery for rectal cancer: current immediate clinical and oncological outcomes. World J Gastroenterol (2014) 20(39):14359–70. 10.3748/wjg.v20.i39.14359 PMC4202365 25339823

[5] Shearer R Gale M Aly OE Aly EH. Have early postoperative complications from laparoscopic rectal cancer surgery improved over the past 20 years? Colorectal Dis (2013) 15(10):1211–26. 10.1111/codi.12302 23711242

[6] Kwak JM Kim SH. Robotic Surgery for Rectal Cancer: An Update in 2015. Cancer Res Treat (2016) 48(2):427–35. 10.4143/crt.2015.478 PMC4843749 26875201

[7] Ahmed J Cao H Panteleimonitis S Khan J Parvaiz A. Robotic vs laparoscopic rectal surgery in high-risk patients. Colorectal Dis (2017) 19(12):1092–9. 10.1111/codi.13783 28644545

[8] Baik SH Kwon HY Kim JS Hur H Sohn SK Cho CH. Robotic versus laparoscopic low anterior resection of rectal cancer: short-term outcome of a prospective comparative study. Ann Surg Oncol (2009) 16(6):1480–7. 10.1245/s10434-009-0435-3 19290486

[9] D’Annibale A Pernazza G Monsellato I Pende V Lucandri G Mazzocchi P. Total mesorectal excision: a comparison of oncological and functional outcomes between robotic and laparoscopic surgery for rectal cancer. Surg Endoscopy (2013) 27(6):1887–95. 10.1007/s00464-012-2731-4 23292566

[10] Miskovic D Ni M Wyles SM Tekkis P Hanna GB. Learning curve and case selection in laparoscopic colorectal surgery: systematic review and international multicenter analysis of 4852 cases. Dis Colon Rectum (2012) 55(12):1300–10. 10.1097/DCR.0b013e31826ab4dd 23135590

[11] Shaw DD Wright M Taylor L Bertelson NL Shashidharan M Menon P. Robotic Colorectal Surgery Learning Curve and Case Complexity. J Laparoendoscopic Advanced Surg Techniq (2018) 28(10):1163–8. 10.1089/lap.2016.0411 29733247

[12] Miskovic D Ahmed J Bissett-Amess R Gómez Ruiz M Luca F Jayne D. European Academy for Robotic Colorectal Surgery (EARCS). European consensus on the standardization of robotic total mesorectal excision for rectal cancer. Colorectal Dis (2019) 21(3):270–6. 10.1111/codi.14502 30489676

[13] Dindo D Demartines N Clavien PA. Classification of surgical complications: a new proposal with evaluation in a cohort of 6336 patients and results of a survey. Ann Surg (2004) 240(2):205–13. 10.1097/01.sla.0000133083.54934.ae PMC1360123 15273542

[14] Jayne D Pigazzi A Marshall H Croft J Corrigan N Copeland J. Effect of Robotic-Assisted vs Conventional Laparoscopic Surgery on Risk of Conversion to Open Laparotomy Among Patients Undergoing Resection for Rectal Cancer: The ROLARR Randomized Clinical Trial. JAMA (2017) 318(16):1569–80. 10.1001/jama.2017.7219 PMC5818805 29067426

[15] Sian TS Tierney GM Park H Lund JN Speake WJ Hurst NG. Robotic colorectal surgery: previous laparoscopic colorectal experience is not essential. J Robotic Surg (2018) 12(2):271–5. 10.1007/s11701-017-0728-7 28721636

[16] Kim IK Kang J Park YA Kim NK Sohn SK Lee KY. Is prior laparoscopy experience required for adaptation to robotic rectal surgery?: Feasibility of one-step transition from open to robotic surgery. Int J Colorectal Dis (2014) 29(6):693–9. 10.1007/s00384-014-1858-2 24770702

[17] Corrigan N Marshall H Croft J Copeland J Jayne D Brown J. Exploring and adjusting for potential learning effects in ROLARR: a randomised controlled trial comparing robotic-assisted vs. standard laparoscopic surgery for rectal cancer resection. Trials (2018) 19(1):339. 10.1186/s13063-018-2726-0 29945673 PMC6020359

[18] Bokhari MB Patel CB Ramos-Valadez DI Ragupathi M Haas EM. Learning curve for robotic-assisted laparoscopic colorectal surgery. Surg Endoscopy (2011) 25(3):855–60. 10.1007/s00464-010-1281-x PMC3044842 20734081

[19] Jiménez-Rodríguez RM Díaz-Pavón JM de la Portilla de Juan F Prendes-Sillero E Dussort HC Padillo J. Learning curve for robotic-assisted laparoscopic rectal cancer surgery. Int J Colorectal Dis (2013) 28(6):815–21. 10.1007/s00384-012-1620-6 23242270

[20] Sng KK Hara M Shin JW Yoo BE Yang KS Kim SH. The multiphasic learning curve for robot-assisted rectal surgery. Surg Endoscopy (2013) 27(9):3297–307. 10.1007/s00464-013-2909-4 23508818

[21] Panteleimonitis S Miskovic D Bissett-Amess R Figueiredo N Turina M Spinoglio G. EARCS Collaborative. Short-term clinical outcomes of a European training programme for robotic colorectal surgery. Surg Endosc. (2020) 12 7. 10.1007/s00464-020-08184-1. Epub ahead of print. PMC8599412 33289055

[22] Panteleimonitis S Popeskou S Aradaib M Harper M Ahmed J Ahmad M. Implementation of robotic rectal surgery training programme: importance of standardisation and structured training. Langenbeck’s Arch Surg (2018) 403(6):749–60. 10.1007/s00423-018-1690-1 PMC6153605 29926187

[23] Aradaib M Neary P Hafeez A Kalbassi R Parvaiz A O’Riordain D. Safe adoption of robotic colorectal surgery using structured training: early Irish experience. J Robotic Surg (2019) 13(5):657–62. 10.1007/s11701-018-00911-0 30536134

[24] Guend H Widmar M Patel S Nash GM Paty PB Guillem JG. Developing a robotic colorectal cancer surgery program: understanding institutional and individual learning curves. Surg Endoscopy (2017) 31(7):2820–8. 10.1007/s00464-016-5292-0 PMC5418100 27815742

