# Supplementary material for: Surgical Complexity and Outcome During the Implementation Phase of a Robotic Colorectal Surgery Program—A Retrospective Cohort Study
Source: Front Oncol. 2021 Feb 16;10:603216. doi: 10.3389/fonc.2020.603216 (PMC7923881; doi:10.3389/fonc.2020.603216)
Supplement: Supplementary file 1 [file DataSheet_1.pdf]

Supplemental Figure 1 - complexity score by Miskovic et al.*

	**Complexity level**			
	I	II	III	IV
BMI (kg/m^2)	<27.5	<30	<30	>30
Resection	Colon	Female pelvic	Male pelvic	Any
Diagnosis				
Cancer[1]	<T3[2]	T3[2]	T3	T4
Inflammatory	None	Uncomplicated[3]	Complicated[4]	Emergency

[1]Preoperative staging (CT, MRI)
[2]Excluding transverse colon, proctocolectomy.
[3]Uncomplicated – eg. diverticular disease withour abscess.
[4]Complicated, diverticular disease with abscess, intra-abdominal fistula, restorative resection for ulcerative colitis

*Miskovic D, Ni M, Wyles SM, Tekkis P, Hanna GB. Learning curve and case selection in laparoscopic colorectal surgery: systematic review and international multicenter analysis of 4852 cases. Diseases of the colon and rectum. 2012;55(12):1300-10.
